# Influence of Smoking on Colonic Gene Expression Profile in Crohn's Disease

**DOI:** 10.1371/journal.pone.0006210

**Published:** 2009-07-15

**Authors:** Ole Haagen Nielsen, Jacob Tveiten Bjerrum, Claudio Csillag, Finn Cilius Nielsen, Jørgen Olsen

**Affiliations:** 1 Department of Gastroenterology, Medical Section, Herlev Hospital, University of Copenhagen, Copenhagen, Denmark; 2 Department of Clinical Biochemistry, Core Unit for Microarray Analyses, Rigshospitalet, University of Copenhagen, Copenhagen, Denmark; 3 Institute of Cellular and Molecular Medicine, Panum Institute, University of Copenhagen, Copenhagen, Denmark; Charité-Universitätsmedizin Berlin, Germany

## Abstract

**Background:**

The development and course of Crohn's disease (CD) is related to both genetic and environmental factors. Smoking has been found to exacerbate the course of CD by increasing the risk of developing fistulas and strictures as well as the need for surgery, possibly because of an interaction between smoking or nicotine on macrophage function and the intestinal microvasculature. Several genes are involved in the pathogenesis of CD, and in this study the gene expression differences of the descending colonic mucosa were investigated in CD (smokers or never smokers) and controls (smokers or never smokers).

**Aim:**

To identify any difference in gene expression of the descending colonic mucosa between smoking and never-smoking CD patients (and controls) by determining genetic expression profiles from microarray analysis.

**Methods:**

Fifty-seven specimens were obtained by routine colonoscopy from the included material: CD smokers (n = 28) or never-smokers (n = 14) as compared to fifteen healthy controls (8 smokers and 7 never-smokers). RNA was isolated and gene expression assessed with Affymetrix GeneChip Human Genome U133 Plus 2.0. Data were analyzed by principal component analysis (PCA), Wilcoxon rank sum test and multiple linear regressions. Real-time (RT) PCR was subsequently applied to verify microarray results.

**Results:**

The PCA analysis showed no intrinsic clustering of smokers *versus* never-smokers. However, when Wilcoxon rank sum test corrected with Q values were performed, six known genes were significantly expressed differently in the inflamed CD smokers as compared to the inflamed CD never-smokers: ring finger protein 138 (RNF138), metalothionein 2A (MT2A) and six transmembrane epithelial antigen of the prostate 3 (STEAP3), SA hypertension-associated homolog, PGM2L1 and KCNJ2. The subsequent RT-PCR-analyses verified, however, that only RNF138, MT2A and STEAP3 were significantly up-regulated in CD smokers in specimens with inflammatory activity of the descending colon.

**Conclusions:**

The present study demonstrates that the genes, RNF138, MT2A, and STEAP3 are differently expressed in the inflamed descending colon of smoking *versus* never-smoking CD patients, which might be of relevance for the poorer clinical course among CD smokers. Many gastroenterologists are still not totally aware of the benefits of smoking cessation in relation to CD, and do not put much effort into getting the patients to quit, therefore more information on the negative effects of smoking, seems warranted.

## Introduction

Crohn's disease (CD) is a chronic inflammatory bowel disease (IBD) which might affect any part of the gastrointestinal tract, causing a wide range of complications including ulceration, fibrostenosis, and fistula development resulting in symptoms like abdominal pain, fever, diarrhea and weight loss during episodes with flare-ups.

Development of the disease is believed to be related to both genetic heritage and various environmental factors [1], such as smoking. While smoking represses activity of ulcerative colitis (UC) [2] it has, however, been shown to exacerbate the course of CD [3], [4], why it is intuitively believed that smoking has different effects on the ileum and colon, though the results of larger studies on this matter have been inconsistent [5], [6]. Furthermore, smoking worsens the course of CD by increasing the risk of developing fistulas and strictures as well as accelerating the need for surgery, probably due to an increased influx of neutrophils into the intestinal mucosa [7], [8]. These detrimental effects of smoking in CD could additionally be related to the nicotine-induced suppression of antimicrobial activity and immune responses by macrophages [9], which might further compound any deficiency in the host response to luminal bacteria. Other components of tobacco smoke, such as oxidizing chemicals, could also be of importance; these unlike nicotine, have prothrombotic effects that may exacerbate microvasculature abnormalities and ischemia [10], [11]. In this way, an individual at risk of CD who smokes might increase the chance of developing the disease, because of the effects of smoking or nicotine on macrophage function and/or the intestinal microvasculature.

DNA microarray technology allows a wide survey of gene expression. It is based on standard hybridization techniques, through scaling up, which allows simultaneous hybridization of thousands of genes fixed on a single solid matrix with mRNA from a single tissue sample [12]. DNA microarrays rely on known nucleotide sequences fixed on a matrix that bind complementary to unknown nucleotide sequences expressed on sample tissues. Since it is believed that there are many genes involved in the pathogenesis of CD, the hypothesized association between the clinical phenotype and the gene expression found in the colonic mucosa of CD patients is likely to be represented by a combinatorial expression pattern of different genes.

In this study, we aimed to identify any gene expression differences between the descending colonic mucosa of smoking and never-smoking CD patients, by determining genetic expression profiles from DNA microarray analysis.

## Methods

### Patients and controls

The present study included 65 humans: 48 CD patients and 17 control subjects (i.e. patients who underwent investigations including colonoscopy, but where all tests were normal). Six CD patients and two control subjects were excluded, as their smoking status could not be established unambiguously. Thus, fifty-seven persons hereof 42 CD patients and 15 control subjects participated in this study (Table 1). A further division of patient groups was based on smoking or never-smoking status. Data from control subjects were used to obtain the normalization of absolute values of mRNA expression.

**Table 1:** Clinical details.

	CD inflamed		CD non-inflamed		Controls		
	Smokers	Never smokers	Smokers	Never smokers	Smokers	Never smokers	
	n = 12	n = 6	n = 16	n = 8	n = 8	n = 7	
Age (years)(mean, range)	34 (18–53)	35 (19–60)	37 (23–58)	32 (20–57)	41 (18–74)	30 (18–51)	
Sex (M/F)	6/6	3/3	5/11	4/4	2/6	1/6	
CDAI	(153<)	(163<)	(>148)	(>143)	-	-	
Years with disease (mean, range)	7 (0–20)	1 (0–3)	13 (2–30)	10 (1–30)	-	-	
Medication:							
Mesalazine	2	1	2	4	-	-	
Azathioprine	1	0	4	1	-	-	
Budesonide	0	0	1	1	-	-	
Systemic glucocorticoids	1	3	1	1	-	-	
Infliximab	2	0	1	0	-	-	
None	4	3	5	1	-	-	
Methotrexate	2	0	2	0	-	-	

CD: Crohn's disease, CDAI: Crohn's Disease Activity Index.

Ongoing treatment of the CD patients was obtained from a questionnaire of the patients themselves and confirmed in medical records, as shown in Table 1. We scored the disease activity by use of Crohn's disease Activity Index (CDAI) [13].

The diagnosis of CD was established on internationally accepted criteria [14], where exclusion criteria excluded people aged over 75 years or below 18 years, where there was clinical evidence of active infections, who had recently used (within 2 weeks) antibiotics, and those who were pregnant or had severe mental illness.

The project was approved by the Scientific Ethics Committee of the Copenhagen Region and was performed in accordance with the Helsinki V Declaration. Informed consent was obtained both written and verbally from all participating patients.

### Tissue Samples

All samples were obtained endoscopically from the descending colon. This part of the intestinal tract was chosen in order to focus on the colonic involvement concerning CD and smoking, to make future comparisons with specimens from patients with ulcerative colitis possible, and to avoid any intersegmental variation in gene expression.

Biopsies for RNA extraction were immediately stabilized in RNA-later (Ambion, Austin, TX, USA). Each tube contained two specimens, which were sampled from the same area, with no more than 2 cm distance in between them. These two specimens were pooled for extraction of RNA. Histopathological evaluation was conducted on biopsies sampled from adjacent areas. This evaluation was performed in an unblinded fashion by staff pathologists, in accordance with well-established criteria and focused on confirming the degree of inflammation [15]. Only biopsies showing both macroscopic and histological signs of inflammation, were considered inflamed. Further, in the non-inflamed patients included into this study, investigations by MRI, x-ray and/or ultrasound did not reveal inflammation in other segments of the gastro-intestinal tract.

### Sample preparation, hybridization, detection and quantification of signals

Intestinal specimens were put immediately in RNA-later at 4°C, where they were kept for 48 h in order to minimize RNA degradation. Specimens were subsequently kept at –20°C until total RNA isolation was initiated. RNA was extracted with Trizol (Invitrogen, Carlsbad, CA, USA) chloroform and precipitated with isopropanol [16]. Further purification was obtained with the RNeasy mini kit (Qiagen, Valencia, CA, USA). Integrity and purity were verified with an Agilent Bioanalyzer (Palo Alto, CA, USA). In accordance with the Affymetrix protocol (Affymetrix, Santa Clara, CA, USA), double-stranded cDNA was synthesized from total RNA, and an in vitro transcription reaction was subsequently performed to produce biotin-labeled cRNA from the cDNA [17]. The cRNA was fragmented and a hybridization mix was prepared, which included the fragmented target, probe array controls, bovine serum albumin (BSA), and herring sperm DNA [17]. In this experiment, the Affymetrix GeneChip Human Genome U133 Plus 2.0 was applied, which contains more than 54.000 probe sets representing approximately 38.500 genes and gene sequences. The hybridized probe array was subsequently stained with fluorescent protein streptavidin-phycoerythrin (SAPE) [18] and scanned with a GeneArray scanner at the excitation wavelength of 488 nm. The amount of light emitted at 570 nm is proportional to the bound target at each location on the probe array.

All significant transcript levels were measured by real-rime reverse transcription (RT)-PCR using the LigthtCycler system (Roche, Manheim, Germany) according to the principles previously described in details [19]. Primers were selected using the Primer Bank resource [20]. Normalization was performed using the transcript level of ribosomal protein L10 (RPL10; Primer Bank ID 5174431a1) as reference.

### Data analysis

A single log2 expression measure for each probe set was calculated from image files (.CEL format) using the robust multi-array analysis (RMA) procedure [21] with quantile normalization implemented in the Affymetrix library for the R statistical environment [22]. Probe sets with background level signal intensities were removed by filtering out probe sets where the lower quantile was below five. 14.378 probe sets remained after filtering. These calculations were performed on the total set of 57 observations and a subset of these observations has previously been analyzed to search for correlations between the colonic gene expression profile and clinical symptoms [17]. The normalized and filtered data were subsequently analyzed by the unsupervised multivariate analysis tool principal component analysis (PCA) using Simca P11 (Umetrics, Umeå, Sweden). PCA was used as it reduces data complexity in a rational way without any prior knowledge of categories so as to determine if any intrinsic clustering or outliers existed within the data set. Five different models were built: Inflamed samples, non-inflamed samples, control samples, CD samples, and all samples. The number of components in the PCA models was determined by using the autofitting option in Simca P11. In addition, Wilcoxon rank sum test was performed comparing smokers *versus* never-smokers in each of four different groups: Controls, CD non-inflamed, CD inflamed, and all observations taken together. All significant results were corrected by calculating the Q value. Wilcoxon rank sum test was also used when comparing real-time RT-PCR results from CD inflamed smokers and never-smokers, and regression analyses were used to test for confounding factors as age, gender, and years with disease. However, because of the number of patients included it would not be sound to use all 4 variables in one model. Instead all variables were tested univariately, and only significant values were tested in a multiple regression model.

The RMA processed data and the image files have been deposited in public databases: ArrayExpress database (CD patients, accession number E-TABM-118) and Gene Expression Omnibus (controls, accession number GSE11831), and the reported microarray data were handled in accordance with the MIAME guidelines.

## Results

**Figure 1:**
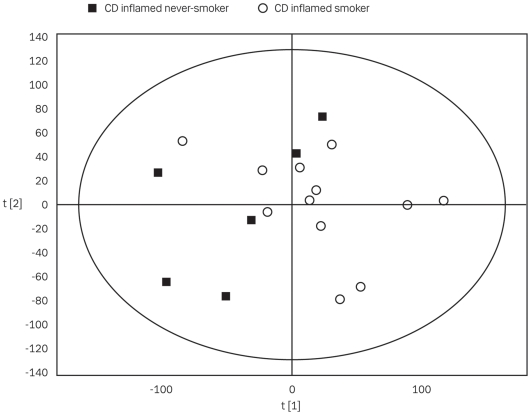
PCA score plot of gene expression from inflamed samples. Gene expression data generated by the Affymetrix HGU U133 Plus 2.0 GeneChips platform and derived from 18 CD patients all with inflamed mucosa were analyzed by PCA. A three-component model was developed explaining a total of 54% (R^2^X) of the variation in the data set and a predictability of 0.22 (Q^2^). The figure shows a score plot with the first two dimensions. Observations are coded according to smoking status. There is no obvious separation of the samples. Analogous PCA models were made for four other datasets (non-inflamed, control, CD and all samples) without any intrinsic clustering.

Twenty-eight of the CD diagnosed patients and eight of the controls entered the smoking group (Table 1). Using PCA the smokers *versus* never-smokers relationship was analyzed. Five different models were constructed by dividing the observations into different sets: Inflamed, non-inflamed, controls, CD and all samples. For each component, score plots were performed and observations colored according to smoking status (see Fig. 1). With no component did an asymmetric distribution of observations of different smoking status become obvious in the score plots of any of the PCA models, nor were any outliers detected. Hence, no correlation between the smoking status and gene expression levels seems to exist, which indicates the absence of a specific “smoking” gene expression pattern. However, a tendency of an asymmetric distribution was observed with CD inflamed smokers *versus* CD inflamed never-smokers (see Fig. 1), and a possible univariate correlation was suspected.

In order to search for such a univariate relationship between the expression level of a single or a few genes and smoking status, a Wilcoxon rank sum test was performed for each gene according to the smoking status. This was carried out in the subgroups for CD inflamed, CD non-inflamed and healthy control samples (data not shown). Twelve known genes were found to be differentially regulated in the CD inflamed smokers group *versus* the CD inflamed never-smokers group (Table 2). However, only six genes had fold changes higher than 1.5, which was chosen as the minimal fold change relevant to report. The expression of these six genes and their potential association with gender and age were tested (linear regression analysis) without any significant correlations. Regarding *years with diagnosis* the patients were divided in two groups: above and below 10 years. It was noted that the 4 patients with diagnosis above 10 years were all smokers, but the association (Fischer's exact test) was not significant (p = 0.2). Two of the six genes were found to be differentially expressed (linear regression) in patients with symptoms for more than 10 years: STEAP family member 3 (STEAP3) and phosphoglucomutase 2-like 1 (PGM2L1). In a multiple regression analysis for STEAP3, the p-values were <0.0001 for smoking status, and 0.1 for diagnosis above 10 years, whereas for PGM2L1 the p-values were 0.0004 for smoking status and 0.02 for diagnosis above 10 years.

**Table 2:** Differential expressed genes on DNA micro-arrays in CD inflamed smokers vs. CD inflamed non-smokers.

ProbeID	Gene Title	Gene Symbol	Wilcoxon rank sum	Q value	Fold changes
212185_x_at	metallothionein 2A	MT2A	0,000108	0,109611	+3
206765_at	potassium inwardly-rectifying channel, subfamily J, member 2	KCNJ2	0,000108	0,109611	+2,4
218424_s_at	STEAP family member 3	STEAP3	0,000108	0,109611	+2,1
229553_at	phosphoglucomutase 2-like 1	PGM2L1	0,000108	0,109611	−1,6
210377_at	SA hypertension-associated homolog (rat)	SAH	0,000108	0,109611	+1,6
218738_s_at	ring finger protein 138	RNF138	0,000108	0,109611	+1,5
225602_at	chromosome 9 open reading frame 19	C9orf19	0,000108	0,109611	+1,4
232165_at	epiplakin 1	EPPK1	0,000108	0,109611	+1,4
223272_s_at	chromosome 1 open reading frame 57	C1orf57	0,000108	0,109611	+1,2
227388_at	tumor suppressor candidate 1	TUSC1	0,000108	0,109611	+1,2
213145_at	F-box and leucine-rich repeat protein 14	FBXL14	0,000108	0,109611	+1
203012_x_at	ribosomal protein L23a	RPL23A	0,000108	0,109611	+0,4

The significance level for the Wilcoxon's rank sum was set at 2×10^−4^.

The Q value is the minimum *false discovery rate* set at 0.15.

Fold Change: up-regulated (+), down-regulated (−) in the smokers.

**Figure 2:**
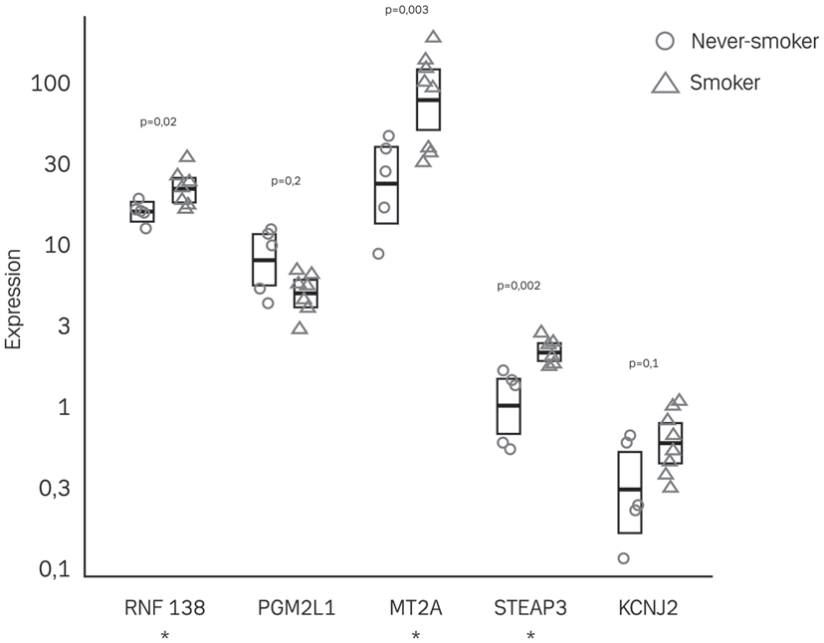
Box plot of RT-PCR results. Real time RT-PCR quantification of genes differentially expressed in inflamed CD smokers and non-smokers. Transcript copy numbers for ring finger protein 138 (RFP138), phosphoglucomutase 2-like 1 (PGM2L1), metallothionein 2A (MT2A), STEAP family member 3 (STEAP3), and potassium inwardly-rectifying channel, subfamily J, member 2 (KCNJ2) were measured in RNA extracted from all inflamed CD biopsy samples. The copy numbers of ribosomal protein L10 were used for normalization between samples. The box border represents the interquartile range, and the horizontal line in the box is the median. *, significant difference between smokers and non-smokers.

In order to verify the DNA micro-array findings, the expression measures of ring finger protein 138 (RNF138), PGM2L1, metallothionein 2A (MT2A), STEAP3, and potassium inwardly-rectifying channel, subfamily J, member 2 (KCNJ2) were quantified by RT-PCR in all included samples from the CD inflamed group. The probe set *210377_at* originally annotated as probing the gene *SA hypertension-associated homolog* in fact probe several genes on chromosome 16 and was therefore omitted for further analysis. Using the Wilcoxon rank sum test this resulted in a significant higher number of RNF138 (two-sided exact test: p = 0.02), MT2A (two-sided exact test: p = 0.03), and STEAP3 (two-sided exact test: p = 0.002) copies in the colonic mucosal biopsies from CD smokers as compared to CD never-smokers, hence verifying the DNA micro-array results (Fig. 2). However, PGM2L1 and KCNJ2 did not reach a statistical significance although the trends in their expression levels were the same in both the RT-PCR and the micro-array analysis.

## Discussion

Smoking is an important environmental factor in IBD, with diametrically opposite effects in UC and CD [23]. Never smoking and having a previous history of smoking increase the risk of active UC [24], whereas smoking increases the risk of developing CD in the general population [24]. In patients with CD, smoking is extremely harmful, and smoking cessation is followed by improvement of the disease [24], [25]. The reason for the opposite association with smoking status is still unclear.

When nicotine has been administered to patients with CD as an enema, some gained benefit, but none deteriorated [23]. Of possible relevance to this observation is the finding that smoking is associated with a higher risk of ileal disease and less colonic involvement [6], and it is believed that smoking has different effects on the ileum and colon [3]. Thus, a larger study of 1,947 IBD patients from 19 European centers of which 630 had CD indicated, that CD patients who smoked were less likely to have colonic involvement (p<0.001), suggesting that smoking might protect the colon from inflammation [6]. Bridger et al [26] defined 339 sibling pairs with IBD, and of those, 89 were discordant for smoking. The sample included 23 pairs who were also discordant for IBD type, and in 21 of these pairs, the smoker developed CD, and the never-smoker UC (OR 10.5 95% CI 2.6–92, p<0.0001). This study additionally suggested that smoking status increase the risk of ileal involvement of CD. However, another study of a French cohort of 978 UC and 688 CD patients, showed that the negative effect of smoking also applied to CD colitis [5].

The etiology of smoking on the course of CD is still unclear, and potentially important mechanisms include cell cycling and apoptosis, immune modulation, permeability and mucous composition, gut vascularity, and perturbation in arachidonate metabolite production [27], [28]. Of the more specified mechanisms by which smoking modulates the immune system is the action of nicotine or the nicotinic acetylcholine receptor α7 subunit [29]. This receptor is expressed on macrophages and mediates the reduction in lipopolysaccharide stimulated TNF-α production, induced by nicotine and vagal nerve stimulation [30]. CD is further characterized by a decreased total radical-trapping antioxidant potential [31], and abnormalities of the microvasculature [32]. Smoking, through increased carbon monoxide concentration, may amplify the impairment in vasodilatation capacity of the chronically inflamed microvessels, resulting in ischemia, and perpetuation of ulceration and fibrosis [10]. The three significantly upregulated and PCR-verified genes identified in the present paper add further explanatory components to the proposed etiology of smoking on the course of CD.

The first significantly up-regulated gene, RNF138, was in the CD inflamed smokers compared to the CD inflamed never-smokers with a fold change of 1.5. RNF138, initially named NARF (NLK-associated RING finger protein) [33], has been shown to ubiquitinate TCF (T-cell factor)/LEF (lymphoid enhancer factor) proteins, which leads to their degradation and hence inhibits Wnt signalling [34]. Wnts are secreted cysteine-rich glycoproteins that act as short-range ligands to locally activate receptor-mediated signalling pathways, and they have been shown to be an essential requirement for normal proliferation and differentiation of the mucosa of the small intestine and colon [35]. The best understood Wnt signalling pathway is the Wnt/β-catenin pathway, which ultimately results in an activation of the DNA bound TCF/LEF transcription factors. Kuhnert et al. [35] injected an adenovirus expressing Dick-kopf-1 (Dkk1), a soluble Wnt antagonist, into adult mice and were able to demonstrate an extensive Dkk1 repression of the TCF/LEF target genes and ablated epithelial proliferation in small intestine and colon accompanied with progressive architectural degeneration with loss of crypts, villi, and glandular structure to the extent of mucosal ulceration and lethality. This conforms with the described role of the Wnt/β-catenin pathway as a “master switch that controls proliferation *versus* differentiation” [36], and an obvious reason why a wide variety of different abnormalities in this pathway have been found to play pivotal roles in oncogenesis, including colorectal cancers [37], [38]. Consequently, the up-regulated RNF138 in the CD inflamed smokers might be one of the factors why smokers with CD have a poorer clinical prognosis than never-smokers with CD, as their colonic mucosal regenerative capabilities are impaired due to the inhibitory effect of RNF138 on the Wnt/β-catenin pathway.

The second upregulated gene by a factor 2.1 was STEAP3 (also named TSAP6 or dudlin-2) which plays a crucial role in tumorogenesis by controlling cell-cycle progression and apoptosis [39], and it might additionally have a pivotal role in regulating vesicular trafficking and secretion [40]. Thus steap proteins appear to play an important role in neoplasia [41]. In addition, STEAP proteins were recently shown to function as metalloreductases. STEAP3 is widely expressed in the body and overexpression stimulates the reduction of iron [42].

The third gene, which was upregulated by a factor 3.0, was MT2A and is involved in induction of apoptosis [43], [44], an important feature in IBD not only for the inflammation but also for malignant transformation [45]. Further, the MT2A protein possess a protection against oxidation injury in particular in inflammatory conditions [46], which might be of importance for the clinical course of CD. Metallothioneins moreover comprise a family of cysteine-rich low molecular weight proteins that form complexes with heavy metal ions such as zinc and copper, but also xenobiotic heavy metals such as mercury, silver and cadmium. Recent data have shown that placental MT2 isoform is upregulated in response to smoking [47]. Cigarette smoke contains about 30 different metal ions including cadmium, zinc, arsene, copper, aluminium, antimony, lead, nickel, chromium and iron [48]. Many metals are reactive and promote oxidative stress and leading to toxic effects and carcinogenesis [48].

Taken together, the upregulation of STEAP3 and MT2 isoform may result from an increased load of metal ions including ferric ions. Apart from local hypersensitivity reactions it is unknown if metalions promote inflammation, but our results indicate that metal ions might play a role in CD.

Two other genes showed significantly altered expression levels on micro-arrays from CD inflamed smokers as compared to the CD inflamed never-smokers: PGM2L1 was 1.6 fold down-regulated, whereas KCNJ2 was upregulated by a factor 2.4. However, a subsequent RT-PCR verification of these genes did not reach a statistical level even though there was a trend towards that PGM2L1 was lower, and KCNJ2 and higher, respectively in mucosal biopsies from CD smokers compared to CD never-smokers.

A range of genes (e.g. CARD15, IBD5 and IL23R) well known to be associated with CD did not show up in this study as these genes are not predominant in the group of CD patients, and because this genome-wide gene association study focused on the smoking phenotype. The lack of a “smoking” gene expression pattern as found in the present paper suggests that no gene expression profile can be attributed to the colonic area of smokers. However, it might be concluded that the genes: RNF138, STEAP3 and MT2A are differently expressed in inflamed descending colonic mucosa of CD smokers *versus* never-smoking CD patients, supporting the notion that the colonic mucosa from that segment of CD patients is in fact affected by the patient's smoking status – a circumstance, which has to be taken into consideration when performing genome-wide gene expression profiles of CD patients and probably also of UC patients. Further, five of CD smokers and one CD never-smoker did receive azathioprine treatment. However, an earlier study of Csillag et al. did not reveal any effect of azathioprine per se on colonic gene expression [49]. It should be noticed, that an even more pronounced difference might be revealed if ileal specimens obtained by ileoscopy were investigated instead of colonic biopsies.

The identification of three genes upregulated in CD smokers, which have a more severe clinical course than non-smokers, might add an important piece in the complex puzzle of the inflammatory process in CD. The question of how smoking alters the expression remains unanswered and needs attention together with functional analyses of the identified genes in future studies. Many gastroenterologists are still not aware of the benefits of smoking cessation in relation to CD, and do not put efforts into getting the patients to quit, therefore more information on the negative effects of smoking, are warranted.
